# A New Method for the Analysis of Bacterial Endotoxins in Ultrapure Paraffin Oil

**DOI:** 10.1155/2014/575246

**Published:** 2014-03-09

**Authors:** Dandan Chen

**Affiliations:** National Institutes for Food and Drug Control, Beijing 100050, China

## Abstract

The paper demonstrates the feasibility of the gel-clot method for the analysis of bacterial endotoxins in water extracts of ultrapure paraffin oil which is a water insoluble oily medical device. Because ultrapure paraffin oil is water insoluble oily liquid, the ultrapure paraffin oil (10 mL) was shaken with 10 mL water for 15 minutes at 2000 rpm, the endotoxin present was extracted to the aqueous phase without interference inhibition/enhancement of the product, the recovery of the endotoxin added to the ultrapure paraffin oil was determined. A validation study confirmed that endotoxins present in ultrapure paraffin oil which is water insoluble liquid medical device pass over into the aqueous phase at concentrations of 20, 10, and 5 EU/mL with recoveries of 94.2% to 111%. So the conclusion is that the gel-clot test is suitable for detecting bacterial endotoxins in ultrapure paraffin oil which is a water insoluble oily medical device.

## 1. Introduction

Ultrapure paraffin oil is used for covering culture solution during artificial fertilization in vitro and micromanipulation of reproduction technique in vitro. The major component of the ultrapure paraffin oil is light paraffin oil. This product has been classified as a class III medical device according to the Chinese Classification Rules of Medical Devices. According to the quality specification of this product the bacterial endotoxin level must be less than 0.25 EU/mL.

The reaction between the Tachypleus Amebocyte Lysate (TAL) and the bacterial endotoxin normally occurs in water solution; that is why water insoluble oily medical devices cannot be analysed directly. There is no reference to this situation in the United States Pharmacopeia [1, 2]. Also no relevant literature has been published on this issue. To this end, the major purpose of this report is to introduce a new method to analyse bacterial endotoxins present in ultrapure paraffin oil and other water insoluble oily medical devices. In this study, the agglutination interference test and the recovery test of soluble extraction of ultrapure paraffin oil have been studied in detail. This is a fundamental and methodological research of bacterial endotoxin analysis for water insoluble oily medical devices with gel-clot test.

## 2. Materials and Methods

### 2.1. Materials

The ultrapure paraffin oil (lot number: 503687) was obtained from VITROLIFE SWEDEN AB. The Tachypleus Amebocyte Lysate with a sensitivity of 0.125 EU/mL (TAL-01, lot number: 1301172) or 0.01~10 EU/mL (lot number: 1203020) and endotoxin-free water (EF-water, lot number: 1207040) were obtained from Zhanjiang Endosafe Biological Ltd. The Tachypleus Amebocyte Lysate with a sensitivity of 0.125 EU/mL (TAL-02, lot number: 130102) was obtained from Zhanjiang Bokang Ocean Biological Co. Ltd. Control standard endotoxins (100 EU per pack, lot number: 150601–201176) were provided by the National Institute for the Control of Food and Pharmaceutical Products, Beijing, China.

### 2.2. Preparation

All equipment and material used in this study were treated at 250°C for more than 1 hour in order to destroy exogenous bacterial endotoxins.

### 2.3. Confirmation of the Labelled Sensitivity of the Tachypleus Amebocyte Lysate

The confirmation was performed according to the requirement of the Chinese Pharmacopoeia 2010, Appendix II, XIE Bacterial Endotoxin Test Regulation [3, 4].

### 2.4. Calculation of the Maximum Valid Dilution Ratio

In order to determine the concentration of possible bacterial endotoxins in the ultrapure paraffin oil the latter was mixed with water and shaken to extract the endotoxin into the aqueous phase. The limit value in the bottom aqueous phase (*L*′) and the endotoxin in the ultrapure paraffin oil have the following relationship:

 formula (1): *L*_ultrapure paraffin oil_ × *V*_ultrapure paraffin oil_ × recovery of the endotoxin  (%) = *L*_water_′ × *V*_water_
 formula (2): *L*_water_′ = (*L* × *V*_ultrapure paraffin oil_ × recovery of the endotoxin)/*V*_water_

According to the regulation of the gel-clot method of the Chinese Pharmacopoeia, the recovery does not need to agree with the calculation of the limit value if the recovery is within 50–200%. Otherwise, the limit value has to be calculated based on the recovery. The limit of ultrapure paraffin oil is 0.25 EU/mL according to the company standard. In the test, 10 mL ultrapure paraffin oil was taken and added to 10 mL endotoxin-free water and shaken for 15 min at 2000 rpm. The limit value in the top aqueous phase is 0.25 EU/mL if the recovery is within 50%–200%. The sensitivity of the marketed Tachypleus Amebocyte Lysate is usually within 0.5–0.03 EU/mL. So the maximum valid dilution ratio of the sample which is corresponding to different sensitivities of Tachypleus Amebocyte Lysate is

(1) $$\begin{array}{c} {\text{MVD}}_{0.25}=\frac{0.25}{0.25}=1;\;\;{\text{MVD}}_{0.125}=\frac{0.25}{0.125}=2;\\ {\text{MVD}}_{0.06}=\frac{0.25}{0.06}=4;\;\;{\text{MVD}}_{0.03}=\frac{0.25}{0.03}=8. \end{array}$$

### 2.5. Interference of the Aqueous Phase Extraction Solution

As the ultrapure paraffin oil is water insoluble oily liquid, its endotoxin must be water-extracted before analysis can be made. In order to determine possible interference factors, 10 mL ultrapure paraffin oil was mixed with 10 mL endotoxin-free water and shaken for 15 min at 2000 rpm. The bottom aqueous phase was then diluted twice with endotoxin-free water followed by endotoxin analysis according to the regulation of the Chinese Pharmacopoeia 2010, Appendix II, “The bacterial endotoxin test sample for the interference test” [5]. Control standard endotoxins were prepared at different concentrations, 0.25, 0.125, 0.06, and 0.03 EU/mL, with endotoxin-free water. The endotoxin-free water and the twice diluted water phase were used as negative controls. Tachypleus Amebocyte Lysate preparations with a sensitivity of 0.125 EU/mL from two different suppliers were applied for comparison.

### 2.6. Recovery of Bacterial Endotoxin in the Aqueous Extract

The extraction rate of endotoxin from the ultrapure paraffin oil to the aqueous phase is defined by the absolute recovery, which must be within 50%–200% in accordance with the requirements of the Chinese Pharmacopoeia. Otherwise the *L*′ = (*L* × *V*_ultrapure paraffin oil_)/(*V*_water_ × recovery) value has to be calculated based on the recovery, to ensure the accuracy of the analysis.

The determination is based on three endotoxin standards at 5, 10, and 20 EU/mL and one ultrapure paraffin oil water extract batch, respectively. 10 mL endotoxin-free water was added to 10 mL of each sample followed by shaking for 15 min at 2000 r/min. The bottom aqueous phase was then diluted 25, 50, 100, and 200 times with EF-water and the recovery was measured using the dynamic turbidimetric analysis method.

### 2.7. Analysis of the Concentration of Bacterial Endotoxin in the Ultrapure Paraffin Oil

10 mL endotoxin-free water was added to 10 mL sample followed by shaking for 15 min at 2000 rpm. The bottom aqueous phase was then analysed for endotoxin according to the gel-clot method of the Chinese Pharmacopoeia 2010, appendix XIE. The endotoxin level must be lower than 0.25 EU/mL. Bacterial endotoxin analysis of twice diluted extracts was performed using a Tachypleus Amebocyte Lysate with a sensitivity of 0.125 EU/mL [6, 7].

## 3. Results

### 3.1. Confirmation of the Labelled Sensitivity of the Tachypleus Amebocyte Lysate

Table 1 shows the results of the confirmation of the sensitivity of two batches of Tachypleus Amebocyte Lysate. The values obtained indicate that the sensitivity of both batches meets the requirement. TAL-01 (*λ* = 0.125) was obtained from Zhanjiang Endosafe Biological Ltd. TAL-02 (*λ* = 0.125) was obtained from Zhanjiang Bokang Ocean Biological Co. Ltd. Plus sign shows a positive result. Minus sign shows a negative result.

### 3.2. Interference Test of the Aqueous Phase Extract

The result of the interference test is shown in Table 2. The results indicate a *E*_*s*_ within 0.5*λ*–2.0*λ* and a *E*_*t*_ within 0.5*E*_*s*_–2.0*E*_*s*_ (*E*_*s*_: bacterial endotoxin of standard, *E*_*t*_: bacterial endotoxin of samples), and it confirms that twice dilution or more will not interfere with the bacterial endotoxin test. Plus sign shows a positive result. Minus sign shows a negative result.

### 3.3. Recovery of Bacterial Endotoxin in the Aqueous Extract

The results of bacterial endotoxin recovery in aqueous extracts of the ultrapure paraffin oil are shown in Table 3.

The data indicate that the recovery of the endotoxin from the ultrapure paraffin oil extract meets the requirement of the gel-clot method of the Chinese Pharmacopoeia which is in the range of 50%–200%.

### 3.4. Analysis of Bacterial Endotoxin of Ultrapure Paraffin Oil

All the analysis results matched the requirements. Result was shown in Table 4.

## 4. Discussion

Because of the water insolubility of the ultrapure paraffin oil, extraction with water is an indispensable part of the bacterial endotoxin test. However, two considerations should be taken into account. Although ultrapure paraffin oil is water insoluble, there may still be some residues in the extracted aqueous phase, so it is important to confirm that the extraction solution will not interfere with the clot between the Tachypleus Amebocyte Lysate and the endotoxin.

Also, since the limit value of the endotoxin in the aqueous phase has the following relationship with the limit value of the endotoxin in the ultrapure paraffin oil, *L*_water_′ = (*L*_ultrapure paraffin oil_ × *V*_ultrapure paraffin oil_ × Percent recovery of the endotoxin)/*V*_water_, the recovery of the endotoxin of the ultrapure paraffin oil must also be considered; otherwise the result of the test will not correlate with the endotoxin in the ultrapure paraffin oil. If the recovery of endotoxin in the ultrapure paraffin oil is within 50%–200% after extraction, then it does not need to be recovered based on the method in Chinese Pharmacopoeia (converted at 100%). If not within 50%–200%, the limit value of the endotoxin in the aqueous phase should be calculated based on the recovery [8]. Due to the different concentrations of endotoxin, their proportion in the ultrapure paraffin oil phase and the aqueous phase and the recovery will be different. This is why the recovery test should be done with different concentrations of endotoxin.

Till present, there are few studies of test methods for bacterial endotoxins in water insoluble oily medical devices. In this study, an interference test was applied to the aqueous phase extracted from the ultrapure paraffin oil to confirm that it did not interfere with the clot formation between the Tachypleus Amebocyte Lysate and the endotoxin. To this end, ultrapure paraffin oil with different endotoxin concentrations was prepared and the recovery was tested using a dynamic turbidimetric method. All results were within 86.8%–96.8% and thus met the requirement of 50%–200% in the Chinese Pharmacopeia. This also indicates that the test method in this study is able to accurately determine the endotoxin concentration in ultrapure paraffin oil by analysis of the aqueous phase extract.

## 5. Conclusions

In this study, we have shown that it is possible to analyse the concentration level of bacterial endotoxin in water extracts of ultrapure paraffin oil using the gel-clot method and verified the limit value of <0.25 EU/mL. At the same time, this study can be regarded as a supplement analysis method for water insoluble oily medical devices to the United States Pharmacopeia chapter number 85 “Analysis of bacterial endotoxin” and chapter number 161 “Blood and other liquid transfusion device and similar medical device.”

## Figures and Tables

**Table 1:** The results of confirmation of the labelled sensitivity.

TAL	Concentration of bacterial endotoxin (EU/mL)				Negative control	*λ*
	0.25	0.125	0.06	0.03		
TAL-01	++++	++++	−−−−	−−−−	− −	0.125
TAL-02	++++	++++	−−−−	−−−−	− −	0.125

**Table 2:** Results of interference test of the ultrapure paraffin oil aqueous phase extract.

TAL	Endotoxin concentration (EU/mL)				Negative control	Results (EU/mL)
	0.25	0.125	0.06	0.03		
TAL-01						
BET	++++	++++	−−−−	−−−−	− −	*E*_*s*_ = 0.125
Sample	++++	++++	−−−−	−−−−	− −	*E*_*t*_ = 0.125
TAL-02						
BET	++++	++++	−−−−	−−−−	− −	*E*_*s*_ = 0.125
Sample	++++	++++	−−−−	−−−−	− −	*E*_*t*_ = 0.125

**Table 3:** Recovery of bacterial endotoxin in the aqueous extracts.

Added bacterial endotoxin (EU/mL)	Measured value (EU/mL)	Recovery (%)
20	19.1	95.5
10	11.1	111
5	4.71	94.2

**Table 4:** Bacterial endotoxin analysis of ultrapure paraffin oil aqueous extracts.

Test sample	Positive product control	Positive control	Negative control
− −	++	++	− −
